# A Review for Consistent Analysis of Hydrogen Permeability through Dense Metallic Membranes

**DOI:** 10.3390/membranes10060120

**Published:** 2020-06-10

**Authors:** Asuka Suzuki, Hiroshi Yukawa

**Affiliations:** 1 Department of Materials Process Engineering, Graduate School of Engineering, Nagoya University, Furo-cho, Chikusa-ku, Nagoya 464-8603, Japan; 2 Department of Materials Design Innovation Engineering, Graduate School of Engineering, Nagoya University, Furo-cho, Chikusa-ku, Nagoya 464-8603, Japan; hiroshi@nagoya-u.jp

**Keywords:** hydrogen permeability, chemical potential, diffusion, pressure-composition isotherm, alloy design, Pd-based alloy, V-based alloy

## Abstract

The hydrogen permeation coefficient (*ϕ*) is generally used as a measure to show hydrogen permeation ability through dense metallic membranes, which is the product of the Fick’s diffusion coefficient (*D*) and the Sieverts’ solubility constant (*K*). However, the hydrogen permeability of metal membranes cannot be analyzed consistently with this conventional description. In this paper, various methods for consistent analysis of hydrogen permeability are reviewed. The derivations of the descriptions are explained in detail and four applications of the consistent descriptions of hydrogen permeability are introduced: (1) prediction of hydrogen flux under given conditions, (2) comparability of hydrogen permeability, (3) understanding of the anomalous temperature dependence of hydrogen permeability of Pd-Ag alloy membrane, and (4) design of alloy composition of non-Pd-based alloy membranes to satisfy both high hydrogen permeability together with strong resistance to hydrogen embrittlement.

## 1. Introduction

Production, purification, storage, and transport of hydrogen gas are essential technologies to use hydrogen as energy effectively [1]. Hydrogen-permeable membranes are important materials for separating and purifying hydrogen gas from a gas mixture [2] produced by the electrolysis [3], steam reforming of hydrocarbon [4], chemical reactions of biomass [5], decomposition reactions of energy carriers [6,7], and so on. Hydrogen-permeable dense metallic membranes can achieve excellent purity of hydrogen gas, which are caused by their characteristic hydrogen permeation mechanism [8]. Hydrogen molecules separate into hydrogen atoms on a surface of a metallic membrane at the feed side (high pressure side) and dissolve into the membrane. Hydrogen atoms diffuse through interstitial sites of metal crystal lattices from the feed side to the permeation side. On a surface of the membrane at the permeation side (low pressure side), hydrogen atoms recombine to form hydrogen molecules. Since other elements (oxygen, nitrogen, carbon, etc.) have a significantly lower diffusion coefficient in metals than hydrogen [9], ultimate hydrogen selectivity (nearly absolute separation) is realized [10].

In most cases of the hydrogen permeation reactions, the diffusion of hydrogen atoms is generally a rate-limiting process. Therefore, hydrogen permeability is commonly described based on the following Fick’s first law. It is noted that the idea of Fick’s law is the driving force for diffusion is the concentration gradient.

(1) $$J=-D\frac{dc}{dx}\;,$$

where *J* is the flux of hydrogen atoms diffusing through a metallic membrane, *D* is the diffusion coefficient of hydrogen, and *dc*/*dx* indicates hydrogen concentration gradient in the permeation direction. It is assumed here that (1) the hydrogen permeation reactions reach the steady state condition and (2) the diffusion coefficient is independent of the hydrogen concentration. Then, Equation (1) is modified as follows.

(2) $$J=D\frac{\Delta c}{L},$$

where Δ*c* is the difference in hydrogen concentrations between the feed and permeation sides, and *L* is the thickness of the membrane. Sieverts’ law expresses the relationship between the hydrogen concentration in metal (*c*) and the pressure of hydrogen gas (*P*) under a condition of dilute hydrogen concentration.

(3) $$c=KP^{0.5},$$

where *K* indicates the hydrogen solubility constant. The exponent of 0.5 means the separation of a hydrogen molecule into two hydrogen atoms. Substituting Equation (3) into Equation (2), the following equation is obtained.

(4) $$J=D\cdot K\frac{\Delta P^{0.5}}{L}=\phi \frac{\Delta P^{0.5}}{L},$$

where Δ*P*^0.5^ is the difference in the square root of hydrogen pressures between the feed and permeation sides. *ϕ* is the hydrogen permeation coefficient, which is described by a product of *D* and *K*. The hydrogen permeation coefficient (*ϕ*) is widely used for evaluating hydrogen permeability of metallic membranes [11,12]. However, a number of researches have reported that the change in hydrogen flux with pressure conditions cannot be described consistently by Equation (4) [13,14,15].

If the hydrogen concentration is non-dilute, the following equation is sometimes used [16].

(5) $$c=K^{\prime }P^{0.5}+\alpha .$$

Substituting Equation (5) into Equation (2), Equation (4) is also obtained. The hydrogen permeability is analyzed only under the conditions in which the hydrogen solubility can be approximated by Equation (5). Therefore, the hydrogen permeability cannot be analyzed and predicted under a wide range of temperatures and pressure conditions.

In order to analyze the hydrogen permeability under a wide range of conditions, Equation (4) is modified into the following power law [13,14,17,18].

(6) $$J=\phi^{\prime }\frac{\Delta P^{n}}{L},$$

where *ϕ*′ is the hydrogen permeation coefficient for the power law. Here, the exponent of 0.5 in Equation (4) is modified into *n* (*n* = 0.5~0.7) in Equation (6). The exponent *n* is optimized for materials and operating temperature and pressure conditions. Then, Equation (6) can analyze the hydrogen permeability of various materials under a wide range of conditions. However, there is no theoretical background for the exponent of *n* not equal to 0.5. Additionally, if *n* changes depending on materials and conditions, *ϕ*′ cannot be used for comparing the hydrogen permeability. 

Recently, some studies have proposed methods for analyzing hydrogen permeability consistently. In this paper, three methods are reviewed: (a) pressure-dependent hydrogen permeation coefficient [15,19,20,21], (b) analysis of hydrogen diffusivity based on thermodynamic factor [22,23,24,25], and (c) consistent description of hydrogen permeability based on hydrogen chemical potential [26]. Four examples of the application of the consistent description of hydrogen permeability are introduced: (a) prediction of hydrogen flux under given conditions, (b) comparability of hydrogen permeability with the hydrogen permeation coefficient for the power law, (c) understanding of the anomalous temperature dependence of hydrogen permeability found recently in Pd-Ag alloy membranes [27], and (d) alloy design of V-based alloy membranes with high hydrogen permeability and strong resistance to hydrogen embrittlement [28]. 

## 2. Methodologies for Analyzing Hydrogen Permeability Consistently

### 2.1. Pressure-Dependent Hydrogen Permeation Coefficient

Hara et al. proposed a pressure-dependent hydrogen permeation coefficient [15,19]. Firstly, assuming that the hydrogen permeation reaction reaches the steady state condition, Equation (1) is modified as follows.

(7) $$J=\frac{1}{L}\int_{c_{2}}^{c_{1}}Ddc=\frac{1}{L}\int_{P_{2}{}^{0.5}}^{P_{1}{}^{0.5}}D\frac{dc}{dP^{0.5}}dP^{0.5},$$

where *c*_1_ and *c*_2_ are the hydrogen concentrations at feed and permeation sides of the membrane, respectively. On the rightmost side, the variable for integration is converted from *c* to *P*^0.5^. Here, the pressure-dependent hydrogen solubility constant (*K*_p_) is defined as follows.

(8) $$K_{p}=\frac{dc}{dP^{0.5}}.$$

**Figure 1:**
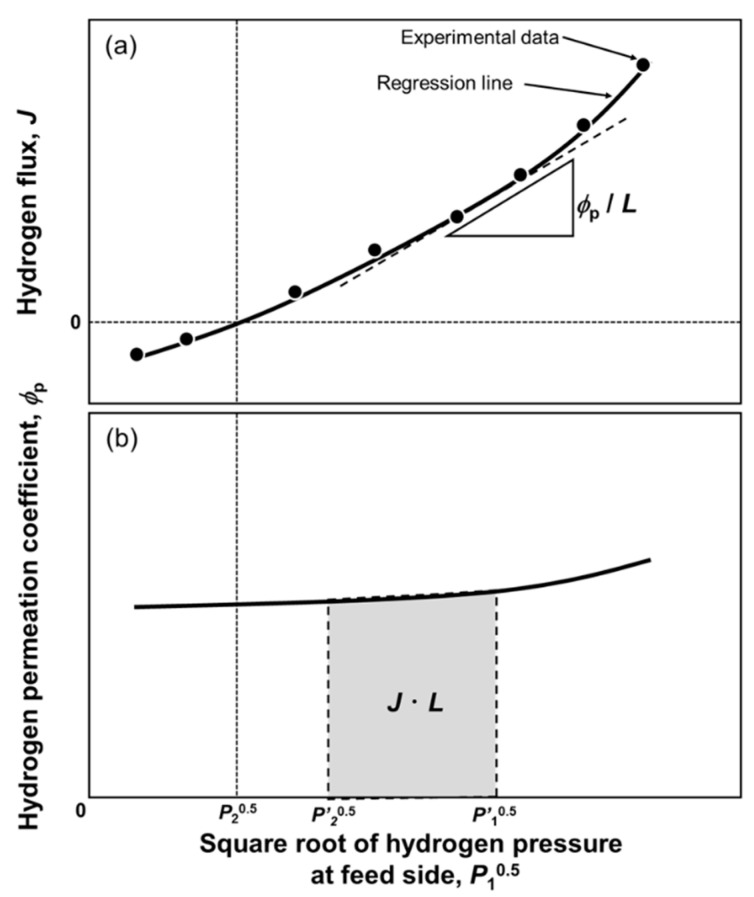
Schematic illustration of changes in (**a**) the hydrogen flux (*J*) and (**b**) pressure-dependent hydrogen permeation coefficient as a function of the square root of hydrogen pressure at the feed side.

Substituting Equation (8) into Equation (7), the following equation is obtained.

(9) $$J=\frac{1}{L}\int_{P_{2}{}^{0.5}}^{P_{1}{}^{0.5}}D\cdot K_{p}dP^{0.5}=\frac{1}{L}\int_{P_{2}{}^{0.5}}^{P_{1}{}^{0.5}}\phi_{p}dP^{0.5},$$

where the *ϕ*_p_ is the pressure-dependent hydrogen permeation coefficient, which is described by the product of *D* and *K*_p_. In order to quantify *ϕ*_p_, a series of hydrogen permeation tests are performed, in which either the hydrogen pressures at the feed or permeation sides are fixed. Figure 1a presents the schematic illustration of the change in the hydrogen flux (*J*) as a function of the square root of hydrogen pressure at the feed side (*P*_1_^0.5^). If the change in *J* cannot be described by Equation (4), *J* increases non-linearly with increasing *P*_1_^0.5^. Then, the experimental data points are regressed by an equation, e.g., a polynomial function. The regression function for *J* is differentiated by *P*_1_^0.5^ to quantify *ϕ*_p_ using the following equation.

(10) $${\phi_{p}|}_{P=P_{1}}=L\frac{dJ}{dP_{1}{}^{0.5}},$$

The change in *ϕ*_p_ with *P*_1_^0.5^ can be drawn like Figure 1b. *ϕ*_p_ is not constant and changes depending on *P*_1_^0.5^. When *J* is predicted under a given pressure condition (*P*′_1_^0.5^ and *P*′_2_^0.5^), *ϕ*_p_ is integrated from *P*′_2_^0.5^ to *P*′_1_^0.5^ (the area of the gray region in Figure 1b). This method can easily analyze the hydrogen permeability under a wide range of pressure conditions. Hara et al. also analyzed the pressure-dependent hydrogen diffusion coefficient and mobility in pure palladium membrane [19]. Caravella et al. analyzed the exponent (*n*) of the power law in Equation (6) based on Equation (10) [20,21].

### 2.2. Analysis of Hydrogen Diffusivity Based on Thermodynamic Factor

Strictly speaking, the driving force for hydrogen diffusion is not the gradient of hydrogen concentration but that of hydrogen chemical potential. Flanagan et al. and Dolan et al. analyzed the hydrogen permeability using the thermodynamic factor based on the hydrogen chemical potential [22,23,24,25]. The diffusion equation based on hydrogen chemical potential is expressed as follows [9].

(11) $$J=-cB\frac{d\mu }{dx},$$

where *B* is the mobility of hydrogen atoms and *dμ*/*dx* indicates the gradient of hydrogen chemical potential in the permeation direction. Equation (11) is simply modified as follows.

(12) $$J=-cB\frac{d\mu }{dc}\cdot \frac{dc}{dx}.$$

The hydrogen chemical potential (*μ*) is a function of the hydrogen concentration (*c*) at a constant temperature. Comparing Equation (12) with Equation (1), the hydrogen diffusion coefficient is described as follows.

(13) $$D=cB\frac{d\mu }{dc}=RTB\cdot \frac{c}{RT}\frac{d\mu }{dc}=D^{*}f_{\mathrm{therm}},$$

where D* is the intrinsic hydrogen diffusion coefficient defined by the Einstein equation (D* = *RTB*) and *f*_therm_ is the thermodynamic factor. In metal-hydrogen systems, the thermodynamic factor as a function of the hydrogen concentration can be obtained from the chemical potential of gaseous hydrogen (*μ*_g_) using the pressure–composition isotherms (PCT curves). In the PCT curves, which are measured under the equilibrium state, the relationship between the chemical potentials of hydrogen atom in metals and gaseous hydrogen are described in the following equation.

(14) $$\mu =\frac{1}{2}\mu_{g}=\frac{1}{2}\left(\mu_{g}^{0}+RT\ln\frac{P}{P^{0}}\right),$$

where *μ*_g_ and *μ*_g_^0^ are the chemical potential and standard chemical potential of gaseous hydrogen, *R* is the gas constant (8.314 J·mol^−1^·K^−1^), *T* is absolute temperature, and *P*^0^ is the standard hydrogen pressure (101325 Pa). The hydrogen concentration is not constant through the membrane during hydrogen permeation (non-equilibrium state). It is assumed here that the chemical potential corresponding to the hydrogen concentration (*c*) at the position (*x*) in the permeation direction can be approximated by Equation (14) (local equilibrium state). Then, the thermodynamic factor is expressed as follows,

(15) $$f_{\mathrm{therm}}=c\frac{d\ln{\left(P/P^{0}\right)}^{0.5}}{dc}=\frac{d\ln{\left(P/P^{0}\right)}^{0.5}}{d\ln c}.$$

**Figure 2:**
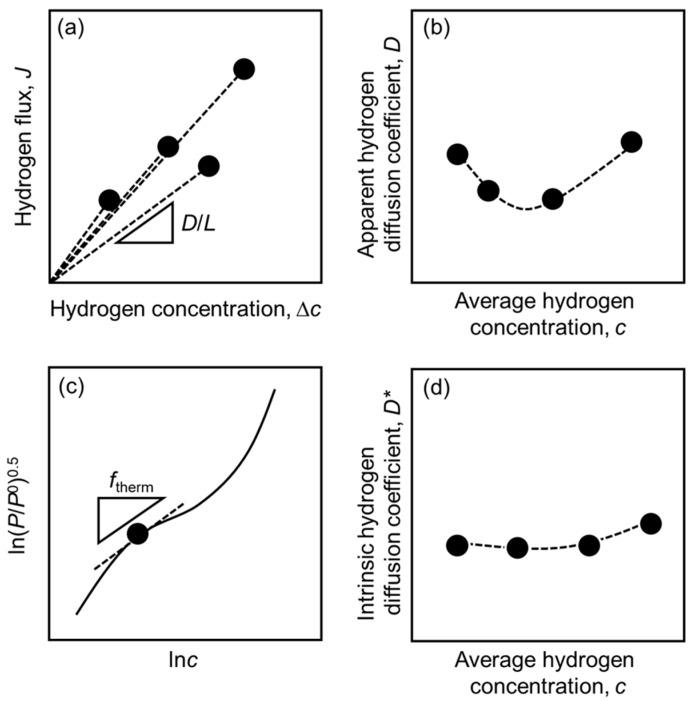
Schematic illustration showing the relationships between (**a**) the hydrogen flux (*J*) and the difference in hydrogen concentration between the feed and permeation sides (Δ*c*), (**b**) apparent hydrogen diffusion coefficient (*D*) and average hydrogen concentration (*c*), (**c**) *d*ln(*P*/*P*^0^)^0.5^ and *d*ln*c*, and (**d**) intrinsic hydrogen diffusion coefficient (D*) and average hydrogen concentration (*c*).

Figure 2a–d shows the schematic illustrations showing methods for analyzing the hydrogen permeability based on Equation (13) and Equation (15). Firstly, change in the hydrogen flux is plotted as a function of the difference in the hydrogen concentration between the feed and permeation sides (Δ*c*) as shown in Figure 2a. Δ*c* are simply estimated from the PCT curves and pressure conditions. The apparent hydrogen diffusion coefficient (*D*) can be estimated from the slopes of the lines passing through the origin and each data point because the slope corresponds to the apparent hydrogen diffusion coefficient (*D*) divided by the thickness of the membrane (*L*) (Equation (2)). Under non-dilute hydrogen concentration condition, *D* is not constant and changes depending on the hydrogen concentration like Figure 2b. The PCT curve is plotted as the relationship between *d*ln(*P*/*P*^0^)^0.5^ and *d*ln*c* like Figure 2c. The gradient of the curve corresponds to the thermodynamic factor (*f*_therm_) according to Equation (15). *D* at each hydrogen concentration is divided by *f*_therm_ to estimate the intrinsic hydrogen diffusion coefficient (D*). Flanagan et al. and Dolan et al. analyzed the D* in Pd-and V-based alloys, respectively [22,23,24,25].

### 2.3. Consistent Description of Hydrogen Permeation Based on Hydrogen Chemical Potential

Suzuki et al. have proposed the consistent description of hydrogen permeability based on hydrogen chemical potential [26], not via Equation (1). When the hydrogen permeation reactions reach the steady state condition, Equation (11) is modified as follows.

(16) $$J=\frac{1}{L}\int_{c_{2}}^{c_{1}}cB\frac{d\mu }{dc}dc.$$

It is assumed here that the mobility of hydrogen atoms is independent of hydrogen concentration. Then, the Equation (16) is modified as follows.

(17) $$J=\frac{B}{L}\int_{c_{2}}^{c_{1}}c\frac{d\mu }{dc}dc,$$

Substituting Equation (14) into Equation (17), the following consistent description of hydrogen permeation is obtained.

(18) $$J=\frac{RTB}{2L}\int_{c_{2}}^{c_{1}}c\frac{d\ln\left(P/P^{0}\right)}{dc}dc=\frac{RTB}{2L}f_{\mathrm{PCT}},$$

where the integral term is defined as the PCT factor (*f*_PCT_) because it can be quantified by analyzing the PCT curves of the material. The term of *d*ln(*P*/*P*^0^)/*dc* corresponds to the slope of the PCT curve, indicating that the hydrogen permeability reflects the shapes of the PCT curves directly. Comparing Equation (18) and Equation (15), the PCT factor corresponds to the integration of the thermodynamic factor from *c*_2_ to *c*_1_. The integration itself is also defined in other studies [22,23] for analyzing the hydrogen diffusion coefficient. Suzuki et al. showed that the hydrogen diffusivity (*B*) and solubility (*f*_PCT_), which contribute to the hydrogen flux (*J*) directly, can be separately analyzed using Equation (18), as stated in Section 3.

**Figure 3:**
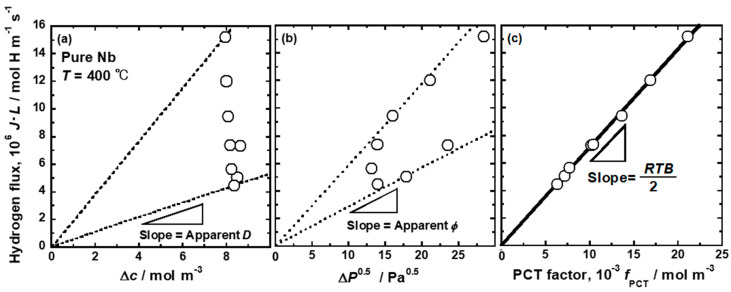
Changes in the hydrogen flux through pure Nb membrane at 400 °C normalized by the inverse of the membrane thickness as functions of (**a**) the differences in hydrogen concentration (∆*c*), (**b**) square root of hydrogen pressures between the feed and permeation sides (Δ*P*^0.5^) and (**c**) PCT factor (*f*_PCT_).

The validity of Equation (18) has been confirmed by the permeation tests for Pd-, Nb-, and V-based alloy membranes [26,27,28,29,30,31,32]. For example, the changes in the hydrogen flux through a pure Nb membrane is measured at 400 °C with different pressure conditions. Figure 3 shows the results of the hydrogen flux normalized by the inverse of the membrane thickness (*J·L*) as functions of (a) the differences in hydrogen concentration (Δ*c*), (b) the difference in square root of hydrogen pressures (Δ*P*^0.5^) between the feed and permeation sides, and (c) the PCT factor (*f*_PCT_) [26]. In Figure 3, the pressure conditions were systematically changed so that the difference in hydrogen concentrations (Δ*c*) between the feed and permeation sides are almost constant of 0.1 (H/M). There are no linear correlations between *J·L* and Δ*c* and between *J·L* and Δ*P*^0.5^ (see Figure 3a,b). The slopes of the lines passing through each data point and the origin correspond to the “apparent” hydrogen diffusion coefficient (*D*) in Figure 3a and hydrogen permeation coefficient (*ϕ*) in Figure 3b. It is evident that the apparent diffusion and permeation coefficients changes depending on the experimental pressure conditions. These results indicate that the assumption to obtain Equations (2) and (4) (the hydrogen diffusion coefficient (*D*) and solubility constant (*K*) are constants) is not valid. On the other hand, there is a linear correlation between *J·L* and *f*_PCT_. The regression line passes through the origin. The slope of the line corresponds to *RTB*/2, indicating that the assumption that the mobility of hydrogen atom (*B*) is independent of hydrogen concentration seems to be valid. Thus, Equation (18) describes the hydrogen permeation consistently well.

It has been recently reported that Equation (18) can be applied to not only pure metals and single-phase alloys but also dual-phase alloys. Zhu et al. showed that there is a linear correlation between *J·L* through Nb_45-x-y_W_x_Mo_y_Ti_27.5_Ni_27.5_ and Nb_56-x_W_x_Ti_23_Co_21_ alloy membranes composed of dual phases (bcc (Nb) and B2 phases) and *f*_PCT_ [30,31]. They stated that the analysis of hydrogen diffusion based on hydrogen chemical potential is more meaningful for dual-phase alloys [31]. The dual-phase alloys exhibit discontinuous profiles of hydrogen concentration at the interfaces of different phases (high concentration in bcc (Nb) and low concentration in B2 phase). It is not appropriate to analyze hydrogen diffusion by Fick’s law considering the concentration gradient as the driving force. However, assuming the local equilibrium state, the chemical potential is a continuous function even at the different phase interfaces.

## 3. Applications of the Consistent Description of Hydrogen Permeability

### 3.1. Prediction of Hydrogen Flux through Pd Membrane under Given Conditions

In this section, precise predictions of hydrogen flux under a given condition by the consistent descriptions are introduced using the results of systematical hydrogen permeation tests for Pd membrane reported by Hara et al. as an example.

**Figure 4:**
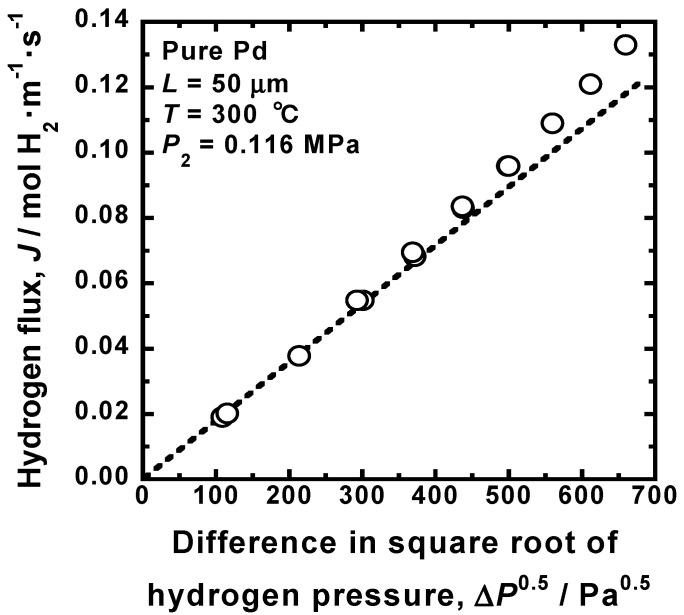
Change in the hydrogen flux through pure Pd membrane at 300 °C as a function of the difference in the square root of hydrogen pressures at the feed and permeation sides [15].

Figure 4 shows the change in the hydrogen flux (*J*) through Pd membrane as a function of the square root of hydrogen pressures between the feed and permeation sides (Δ*P*^0.5^) at 300 °C, reported by Hara et al. [15]. The hydrogen pressure at the permeation side is fixed at 0.116 MPa. There seems to be almost linear correlation between *J* and Δ*P*^0.5^. When the data points are regressed by Equation (4), the hydrogen permeation coefficient is estimated to be approximately 9.65 × 10^−9^ mol H_2_·m^−1^·s^−1^·Pa^−0.5^. The broken line passes through the origin and the data points at relatively low hydrogen pressures. The data points at higher hydrogen pressures deviate from the broken line. Strictly speaking, the hydrogen flux does not change following Equation (4).

**Figure 5:**
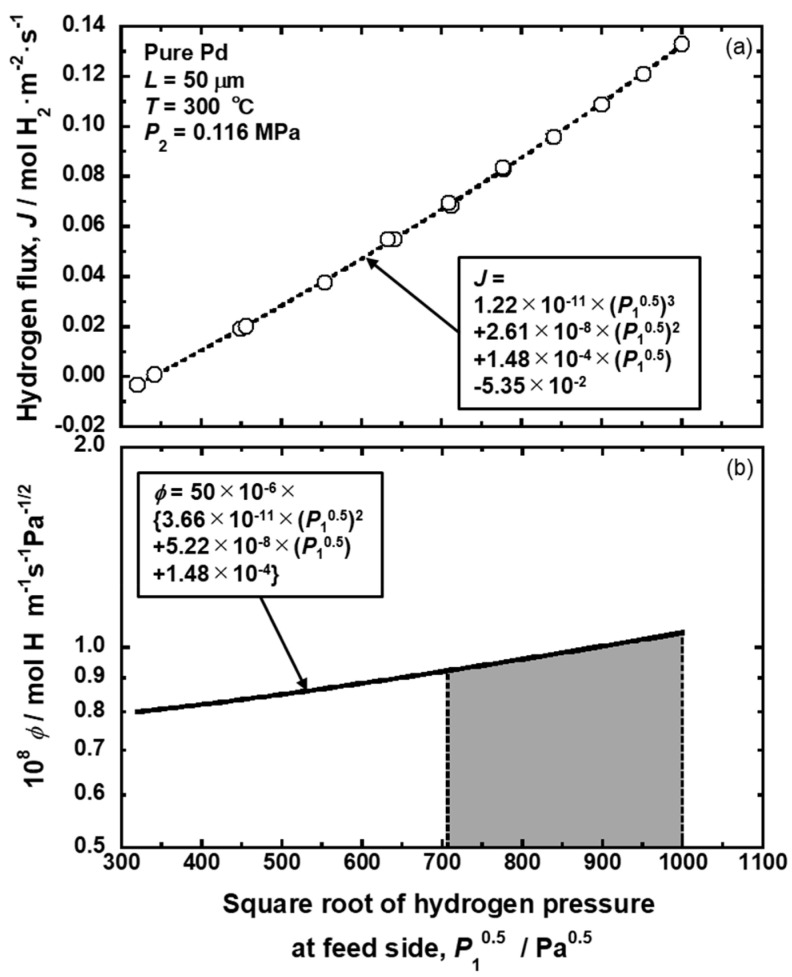
Changes in (**a**) the hydrogen flux and (**b**) the pressure-dependent hydrogen permeation coefficient for pure Pd membrane as a function of the square root of hydrogen pressure at the feed side [15].

When the pressure-dependent hydrogen permeation coefficient is used, changes in the hydrogen flux are described as a function of the square root of hydrogen pressure at the feed side, as shown in Figure 5a [15]. The data points in Figure 5a are regressed by the following third-polynomial function.

(19) $$J=a_{3}{\left(P_{1}{}^{0.5}\right)}^{3}+a_{2}{\left(P_{1}{}^{0.5}\right)}^{2}+a_{1}(P_{1}{}^{0.5})+a_{0},$$

where *a_i_* is the regression coefficient. In the case of Figure 5a, *a*_3_, *a*_2_, *a*_1_, and *a*_0_ are 1.22 × 10^−11^, 2.61 × 10^−8^, 1.48 × 10^−4^, and −5.35 × 10^−2^, respectively. Substituting Equation (19) into Equation (10), the pressure-dependent hydrogen permeation coefficient is expressed as follows and described in Figure 5b.

(20) $${\phi_{p}|}_{P=P_{1}}=L\left\{3a_{3}{\left(P_{1}{}^{0.5}\right)}^{2}+2a_{2}(P_{1}{}^{0.5})+a_{1}\right\}.$$

**Figure 6:**
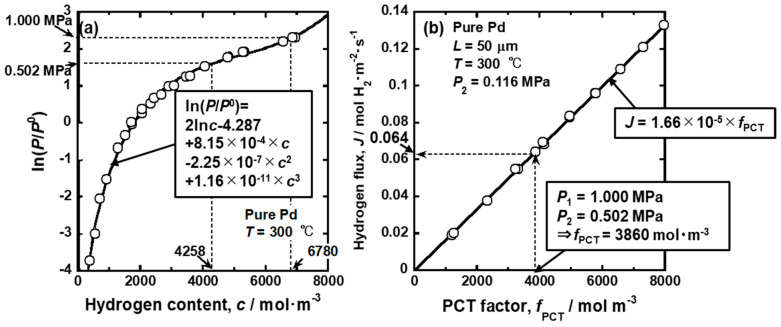
(**a**) pressure–composition isotherm (PCT curve) for pure Pd at 300 °C and (**b**) change in the hydrogen flux through pure Pd membrane at 300 °C in Figure 4 replotted as a function of the PCT factor.

On the other hand, when the PCT factor is analyzed, the PCT curve at the same temperature as the permeation tests is measured, as shown in Figure 6a. The PCT curve is regressed in the following equation

(21) $$\ln\left(P/P^{0}\right)=2\ln c+m_{0}+\sum_{i=1}m_{i}c^{i},$$

where *m_i_* is the regression coefficient. The first and second terms correspond to Sieverts’ law, and third terms approximate the deviations from Sieverts’ law as a polynomial function. Substituting Equation (21) into Equation (18), the PCT factors at each condition are quantified. Figure 6b shows the change in the hydrogen flux in Figure 4 replotted as a function of the PCT factor. There is a linear correlation between the hydrogen flux and the PCT factor. The regression line passes through the origin, indicating that the hydrogen permeation reaction takes place following Equation (18).

When Equation (20) is integrated or the PCT factor is estimated under different pressure conditions, the hydrogen flux can be estimated. For example, the hydrogen pressures at the feed and permeation sides are set at 1.000 and 0.502 MPa. In the case of the pressure-dependent hydrogen permeation coefficient, the hydrogen flux under this condition is estimated as the area of the gray region in Figure 5b. When the PCT factor is analyzed, the hydrogen concentrations at the feed and permeation sides (*c*_1_ and *c*_2_) are estimated to be 6780 and 4258 mol·m^−3^, respectively, from the PCT curve and the pressure condition, as shown in Figure 6a. By integrating *c*×*d*ln(*P*/*P*^0^)/*dc* in Equation (18) from 4258 to 6780 mol·m^−3^, the PCT factor under this condition is quantified as about 3860 mol·m^−3^. Then, the hydrogen flux can be estimated like the dotted arrow in Figure 6b.

**Figure 7:**
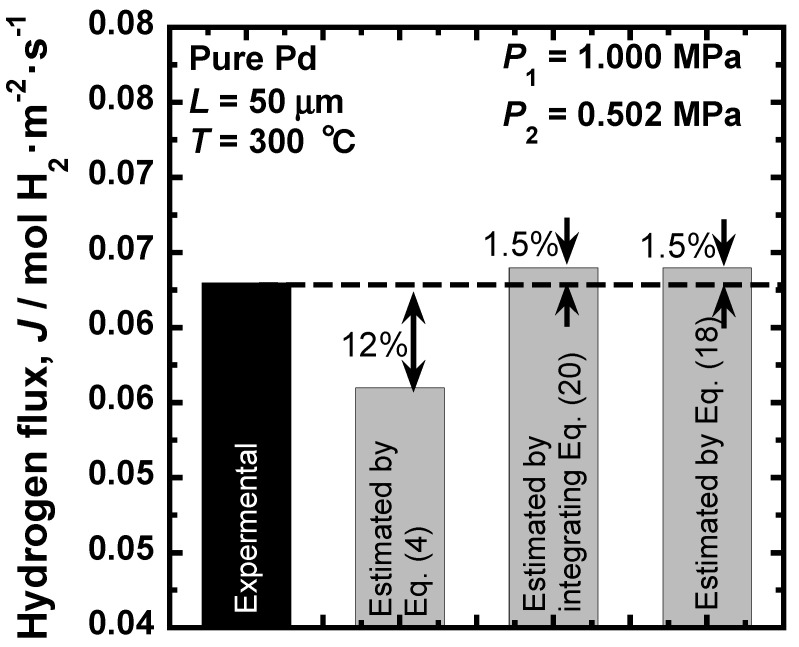
Comparison of the experimental and estimated hydrogen fluxes through pure Pd membrane at 300 °C under a pressure condition of 1.000 MPa at the feed side and 0.502 MPa at the permeation side. The estimations were carried out using the conventional hydrogen permeation coefficient, the pressure-dependent hydrogen permeation coefficient, and the PCT factor.

Figure 7 shows a comparison between experimental and estimated hydrogen fluxes. The experimental hydrogen flux is approximately 6.3 × 10^−2^ mol H_2_·m^−2^·s^−1^ [15]. The hydrogen flux estimated by substituting the value of hydrogen permeation coefficient, 9.65 × 10^−9^ mol H_2_·m^−1^·s^−1^·Pa^−0.5^, into Equation (4) is approximately 5.6 × 10^−2^ mol H_2_·m^−2^·s^−1^. The error from the experimental value is about 12%. On the other hand, the hydrogen fluxes estimated by integrating Equation (20) and by Equation (18) is approximately 6.4 × 10^−2^ mol H_2_·m^−2^·s^−1^. The errors from the experimental value are about 1.5% in both cases, about one order smaller than the value estimated by the conventional description. Thus, the consistent descriptions of hydrogen permeability are useful for predicting the hydrogen flux precisely.

### 3.2. Comparability of Hydrogen Permeability

As mentioned in Section 1, the hydrogen permeability is often analyzed by Equation (6) (power law). The hydrogen permeation coefficient for the power law cannot be compared with the hydrogen permeation coefficient in Equation (4) because the exponent (*n*) is different from 0.5. However, the consistent descriptions of hydrogen permeability overcome the problem and make it possible to compare hydrogen permeation coefficients for the power law with different *n*. In this section, the application of the pressure-dependent hydrogen permeation coefficient is explained.

Substituting Equation (6) into Equation (10), the following equation is obtained [15].

(22) $${\phi_{p}|}_{P=P_{1}}=2n\phi^{\prime }{\left(P_{1}{}^{0.5}\right)}^{2n-1},$$

**Figure 8:**
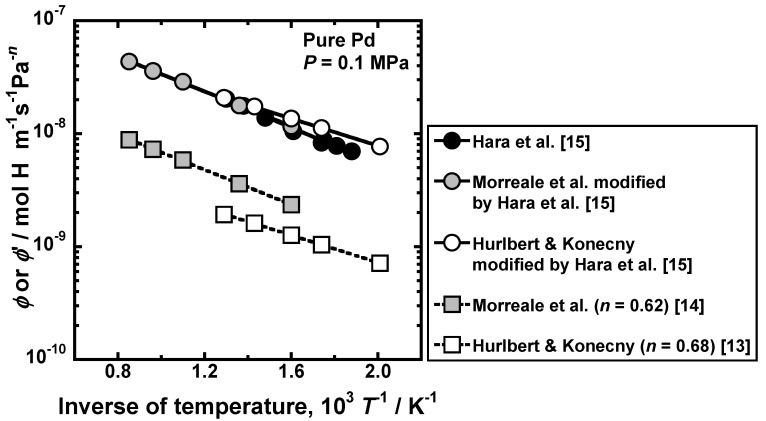
Arrhenius plot of the hydrogen permeation coefficient for the power law reported in the literature [13,14] and the pressure-dependent hydrogen permeation coefficient at 0.1 MPa [15].

Equation (22) indicates that *ϕ*′ can be easily converted into *ϕ*_p_. For example, Hurlbert and Konecny [13] and Morreale et al. [14] reported the hydrogen permeation coefficients for the power law of *n* = 0.68 and 0.62, respectively, which are shown by open and gray square symbols in Figure 8. When the exponent (*n*) is different, the hydrogen permeation coefficients for the power law are significantly different even at the same temperature. Hare et al. quantified the pressure-dependent hydrogen permeation coefficients at 0.1 MPa from these results by Equation (22), which are indicated by the open and gray circle symbols in Figure 8. The black symbols indicate the pressure-dependent hydrogen permeation coefficient at 0.1 MPa reported by Hara et al. These data are almost comparable, indicating that even the hydrogen permeation coefficient for the power law with different *n* can be compared using the pressure-dependent hydrogen permeation coefficient.

### 3.3. Understanding of Reverse Temperature Dependence of Hydrogen Permeability through Pd-Ag Alloy Membrane

Pd-Ag alloy membranes are widely used as hydrogen-permeable membranes because of their high hydrogen permeability, excellent hydrogen selectivity, and strong resistance to oxidation and hydrogen embrittlement. It has been considered that the hydrogen permeability of Pd-based alloy membranes cannot be used practically below 300 °C, as the hydrogen diffusivity as well as the hydrogen permeability decrease with a decreasing operation temperature.

**Figure 9:**
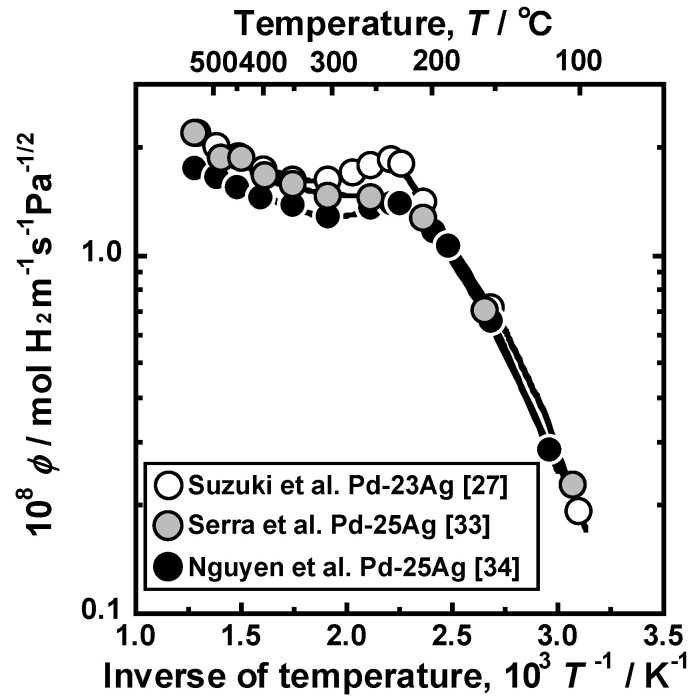
Arrhenius plot of hydrogen permeation coefficient (*ϕ*) of Pd–23 mol%Ag alloy membrane [27]. The temperature dependence of hydrogen permeation coefficients of Pd−25 mol%Ag reported in the literature [33,34] is also shown in the figure.

However, the reverse temperature dependence of the hydrogen permeability has been reported recently in Pd-Ag alloy membranes [27]. Figure 9 shows the Arrhenius plot of the hydrogen permeation coefficient of Pd−23 mol%Ag and Pd−25 mol%Ag alloy membranes [27,33,34]. Above 300 °C, the logarithmic hydrogen permeation coefficient decreases almost linearly with increasing the inverse of temperature. However, as reported in Suzuki et al. [27] and Nguyen et al. [34], the hydrogen permeation coefficient inversely increases with decreasing temperature below 250 °C, and a peak is observed at around 180 °C. The hydrogen permeation coefficient reported by Serra et al. [33] does not have a peak but shows a shoulder, although the authors did not mention it at all. These results imply a possibility of the utilization of Pd-based alloy membranes for low temperature application. For example, according to Suzuki et al. [27], the hydrogen permeation coefficient at the peak temperature, i.e., 180 °C, is comparable with the value obtained at 400 °C, indicating that the operating temperature of the membrane can be lowered by about 220 °C while maintaining high hydrogen permeability.

Nguyen et al. stated that the reverse temperature dependence in Figure 9 may be caused by the balance of hydrogen diffusivity and solubility [34]. Okazaki et al. reported a similar temperature dependence of hydrogen flux through Pd-5Ag, 10Ag, and 20 Ag alloy membranes [35]. They discuss that the anomalous temperature dependence is related to the α-α′ phase transition, which is predicted from the equilibrium phase diagram for Pd-H binary system. [35]. However, in the case of Pd-23~25 mol%Ag alloy membranes, the α-α′ phase transition is not the reason for this anomalous temperature dependence because the addition of Ag into Pd suppresses the α-α′ phase transition, and the critical temperature becomes approximately 20 °C [27]. Additionally, the rate limiting process of hydrogen permeation reaction is hydrogen diffusion through the metal membrane, and it is conformed to be unchanged in the wide temperature range of 22~500 °C [27]. The diffusion-controlled hydrogen permeation in Pd and Pd-Ag alloy membranes at 150 and 200 °C were also reported by Flanagan et al. [22,36]. On the other hand, the surface effects on hydrogen permeability were also pointed out [14,37]. There are two possible reasons why the rate-limiting process is the hydrogen diffusion under a wide temperature range: (a) sufficient thick membrane and (b) surface treatment. In the reviewed study [27], membranes with the thicknesses of 30 and 60 μm were used. If the thickness is below 30 μm, the surface effects might be exhibited. Additionally, in the study [27], so-called “air-treatment”, which is high temperature oxidation in air and subsequent reduction in hydrogen, were applied to the membrane. It is reported that the air-treatment activates the surface of the membrane [38], resulting in diffusion-controlled hydrogen permeation reaction under a wide range of temperatures.

**Figure 10:**
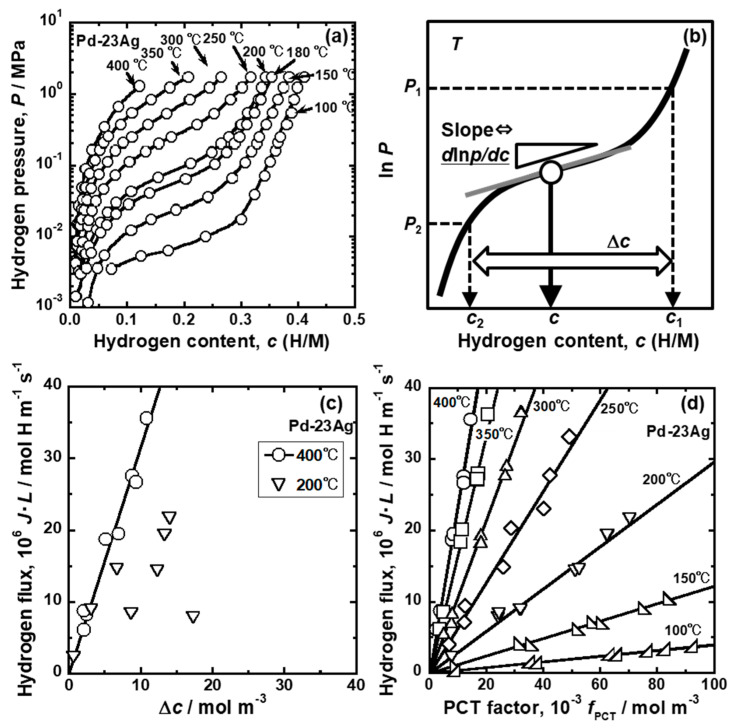
(**a**) Pressure–composition isotherms (PCT curves) for Pd-23 mol%Ag alloy, (**b**) schematic illustration of a PCT curve showing how to estimate the difference in hydrogen concentrations between feed and permeation sides (Δ*c*) and the PCT factor (*f*_PCT_), and (**c**,**d**) changes in the hydrogen flux normalized by the inverse of membrane thickness as functions of (**c**) Δ*c* and (**d**) *f*_PCT_ [27], with copyright permission from Japan Institute of Metal and Materials (JIM).

Figure 10 presents (a) PCT curves of the Pd-23 mol%Ag alloy in the range of 100~400 °C [27], (b) schematic illustration of a PCT curve showing how to estimate the difference in hydrogen concentrations between the feed and permeation sides (Δ*c*) and the PCT factor (*f*_PCT_), (c, d) changes in hydrogen flux for Pd-23 mol%Ag normalized by the inverse of membrane thickness (*J·L*) as functions of Δ*c* and *f*_PCT_ [27]. Using the PCT curves shown in Figure 10a, Δ*c* can be simply estimated from the hydrogen pressures at the feed and permeation sides (*P*_1_ and *P*_2_) as shown in Figure 10b. The values of *f*_PCT_ can also be estimated from the PCT curves and the pressure conditions (Figure 10b). The pressure conditions provide hydrogen concentrations at feed and permeation sides (*c*_1_ and *c*_2_), which are used for the integral interval in Equation (18). The products of hydrogen concentration (*c*) and the slope of the PCT curves (*d*ln(*P/P*^0^)/*dc*) are integrated from *c*_2_ to *c*_1_ to quantify *f*_PCT_. In order to estimate *d*ln(*P/P*^0^)/*dc*, the PCT curves are regressed by Equation (21). As shown in Figure 10c, there is a linear correlation between *J·L* and Δ*c* at 400 °C. However, the data points are scattered and do not show any linear correlation at 200 °C. These results indicate that the hydrogen permeability cannot be analyzed consistently in a wide range of temperatures by Equation (2), and also by Equation (4) based on Equation (2).

On the other hand, there is a linear correlation between *J·L* and *f*_PCT_ in a wide temperature range of 100~400 °C (Figure 10d). The regression lines pass through the origin, indicating that the hydrogen permeation reaction takes place following Equation (18). Thus, Equation (18) permits analyzing the hydrogen permeability consistently even at low temperatures at which the anomalous temperature dependence of hydrogen permeability is exhibited.

**Figure 11:**
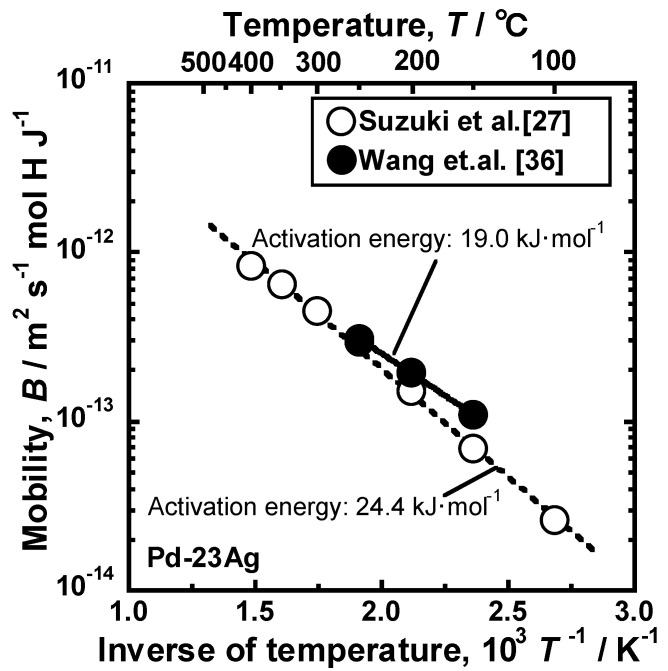
Arrhenius plot of the mobility (*B*) for hydrogen diffusion in Pd–23 mol%Ag alloy [27]. The intrinsic hydrogen diffusion coefficients reported by Wang et al. [39] are modified into the mobility for hydrogen diffusion and plotted in the figure, with copyright permission from Japan Institute of Metal and Materials (JIM).

In Figure 10d, the slopes of the regression lines correspond to *RTB*/2, according to Equation (18), indicating that the mobility of hydrogen atoms (*B*) can be quantified. Figure 11 shows the Arrhenius plot of the mobility of hydrogen atoms [27]. For comparison, the results of the intrinsic hydrogen diffusion coefficient reported by Wang et al. [36] are converted into the mobility for hydrogen diffusion and shown in the figure. The logarithmic mobility of hydrogen atoms decreases almost linearly with increasing the inverse of temperature. The values reported by Suzuki et al. are slightly lower than the ones given by Wang et al. [36] but they are almost comparable. The activation energy for hydrogen diffusion is estimated to be approximately 24.4 and 19.0 J·mol^−1^, respectively. Here, it is important to note that the logarithmic mobility of hydrogen atoms decreases monotonically even at the temperature at which the anomalous temperature dependence of the hydrogen permeation coefficient is exhibited.

**Figure 12:**
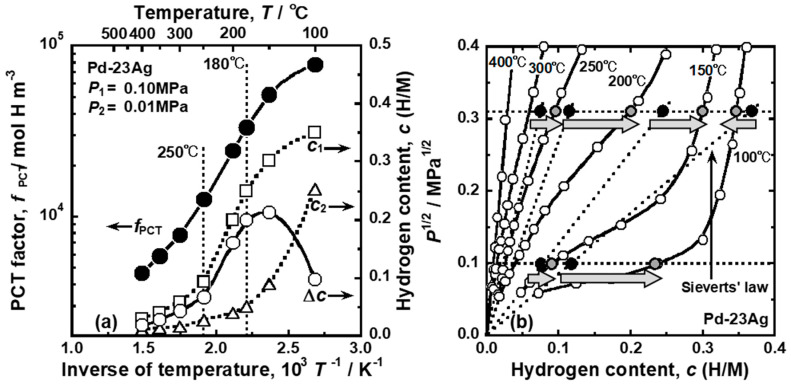
(**a**) Temperature dependence of the PCT factor (*f*_PCT_), the hydrogen concentration at each feed and permeation side of the membrane (*c*_1_ and *c*_2_), and their difference (Δ*c*) when the pressure condition of the feed and permeation sides are fixed to be 0.10 and 0.01 MPa, respectively [27]. (**b**) PCT curves of Pd-23 mol%Ag alloy at low pressure range [27], with copyright permission from Japan Institute of Metal and Materials (JIM).

Figure 12 presents (a) changes in the PCT factor, hydrogen concentrations at feed and permeation sides (*c*_1_ and *c*_2_), and their difference (Δ*c*) as a function of the inverse of temperature under a pressure condition of 0.10 MPa at the feed side and 0.01 MPa at the permeation side, and (b) the PCT curves of Pd-23 mol%Ag alloy at low pressure range [27]. The PCT factor increases along a sigmoid curve with decreasing temperature (Figure 12a). Above 300 °C, the PCT factor increases almost linearly with decreasing temperature. However, at around 250 °C, the hydrogen concentration at the feed side increases drastically, which increases the integral interval (Δ*c*) and hydrogen concentration (*c*) in Equation (18), resulting in a significant increment in the PCT factor. At around 180 °C, hydrogen concentration at the permeation side starts to increase drastically while the one at the feed side increases slightly with decreasing temperature. As a result, the integral interval (Δ*c*) in Equation (18) decreases, resulting in a moderate increase in the PCT factor.

The temperature dependence of the PCT factor causes the reverse temperature dependence of the hydrogen permeation coefficient from 250 to 180 °C, as shown in Figure 9. The changes in hydrogen concentrations, which cause the temperature dependence of the PCT factor, is understood in view of the PCT curves shown in Figure 12b. In this figure, *P*^0.5^ is used for the vertical axis. Above 300 °C, there are linear correlations between *P*^0.5^ and hydrogen concentration (*c*), indicating that the hydrogen solubility is described by Sieverts’ law. At 250 °C, hydrogen concentration at the feed side is higher than the value estimated from Sieverts’ law, which corresponds to the significant increase in *c*_1_ and the integral interval (Δ*c*) in Figure 12a. At 100 °C, the hydrogen concentration at the permeation side also becomes higher than the value estimated from Sieverts’ law. Simultaneously, the hydrogen concentration at the feed side becomes lower than the value estimated from Sieverts’ law due to the steep slope of the PCT curve. These deviations from Sieverts’ law result in the decrease in the integral interval (Δ*c*) in Figure 12a.

Based on the discussion above, it is revealed that the anomalous temperature dependence of hydrogen permeation coefficient shown in Figure 9 is caused by the deviation of hydrogen solubility from Sieverts’ law. Even if Sieverts’ law cannot describe hydrogen solubility, hydrogen permeability can be analyzed consistently by Equation (18). Equation (18) also provides a way to determine silver concentrations in Pd-Ag alloy to make the anomalous temperature dependence more significant, as reported in the literature [27]. It is important to investigate the temperature dependence of hydrogen permeability in other alloys in the future.

### 3.4. Design of Non-Pd-Based Alloy Membranes with High Hydrogen Permeability Together with Strong Resistance to Hydrogen Embrittlement

In this section, an example of the application of the consistent description will be reviewed, focusing on the alloy design of V-Fe alloy membrane with high hydrogen permeability together with strong resistance to hydrogen embrittlement [28].

Although Pd-based alloy membranes are promising materials for hydrogen separation and purification, non-Pd-based alloy membranes are strongly required in order to reduce material costs and improve hydrogen permeability. Group 5 metal (Nb, V, and Ta)-based alloy membranes have been recently developed [39,40,41,42] because they are less expensive and exhibit higher hydrogen permeability than Pd [43]. However, Group 5 metals exhibit poor resistance to hydrogen embrittlement, which is a large barrier to the practical use of them. Matsumoto et al. investigated the mechanical properties of Group 5 metals by the in situ small punch (SP) method under hydrogen atmosphere at high temperature [44,45]. It is revealed that the SP absorption energy decreases drastically at a hydrogen concentration of 0.2~0.25 (H/M). The threshold hydrogen concentration is defined as the “ductile-to-brittle transition hydrogen concentration (DBTC)”. In order to prevent hydrogen embrittlement, the hydrogen concentration must be suppressed below the DBTC.

**Figure 13:**
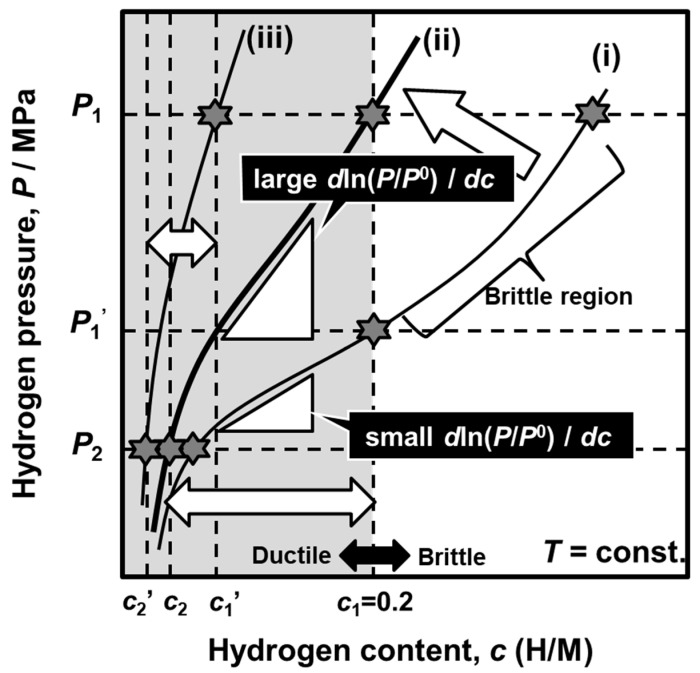
Schematic illustration of PCT curves showing the concept for alloy design [28,32], with copyright permission from Japan Institute of Metal and Materials (JIM).

Here, a concept for alloy design to enhance hydrogen flux while suppressing hydrogen concentration below the DBTC will be discussed in view of the PCT factor. Figure 13 shows the schematic illustration of PCT curves showing the concept for alloy design [28]. Based on Equation (18), there are three ways to enhance hydrogen flux: (1) increase the difference of hydrogen concentrations between the feed and permeation sides (integral interval, *c*_2_~*c*_1_), (2) increase the hydrogen concentration (*c*), i.e., operate under a high hydrogen concentration condition, and (3) increase the slope of the PCT curve (*d*ln(*P*/*P*^0^)/*dc*). For example, when a metal (or an alloy) with the PCT curve (i) in Figure 13 is used as a membrane under pressures of *P*_1_ and *P*_2_, large integral interval and high hydrogen concentration are obtained (the ways (1) and (2) are satisfied). However, hydrogen concentration exceeds the DBTC largely, resulting in the failure of the membrane due to severe hydrogen embrittlement. In order to avoid the hydrogen embrittlement, the hydrogen pressure at the feed side needs to be lowered to *P*_1_′. A better way is to shift the PCT curve to the upper left region like the curve (ii) in some way, for example, by alloying. Then, not only the hydrogen concentration is suppressed below the DBTC, but also the slope of the PCT curve (*d*ln(*P*/*P*^0^)/*dc*) becomes steeper (the way (3) is satisfied) to enhance the hydrogen flux. When the PCT curve is shifted further to the curve (iii), the slope of the PCT curve (*d*ln(*P*/*P*^0^)/*dc*) becomes much steeper but the integral interval (*c*_2′_~*c*_1′_) and hydrogen concentration become significantly small, leading to a decrement in the PCT factor [32]. In order to enhance high hydrogen flux while preventing hydrogen embrittlement, an alloy with appropriate hydrogen solubility like the PCT curve (ii) under given temperature and pressure conditions needs to be designed.

**Figure 14:**
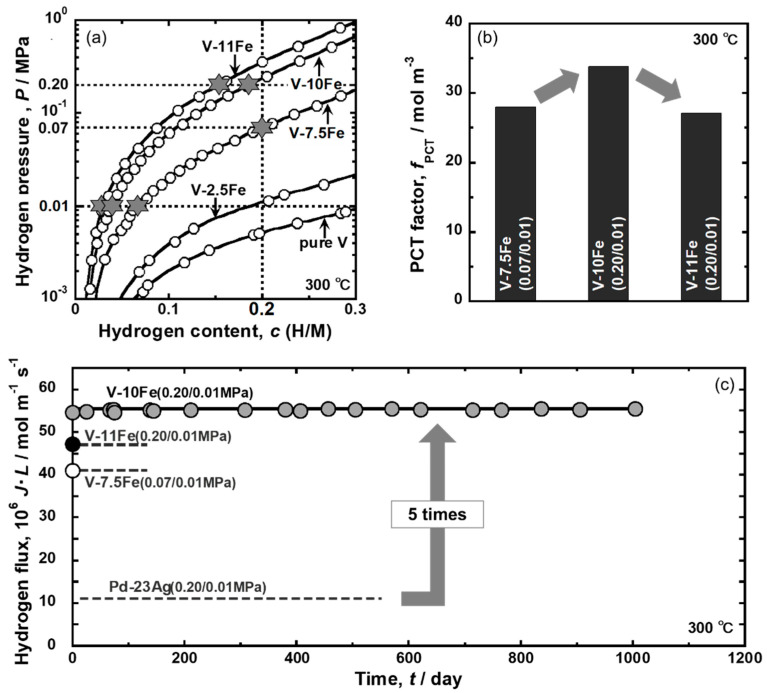
(**a**) PCT curves of pure V [47] and V-Fe alloys at 300 °C, (**b**) PCT factors for each alloy at each condition represented by star symbol (☆) in (**a**), and (**c**) time dependence of hydrogen flux normalized by the inverse of membrane thickness (*J·L*) for Pd-27 mol%Ag-coated V-10 mol%Fe alloy membranes at 300 °C. The experimental results for V-7.5 and V-11 mol%Fe alloy membranes under the conditions represented by the star symbols in (**a**) and the estimated value for Pd-23 mol%Ag alloy under the same condition as V-10 mol%Fe alloy are also shown in the figure [28].

As an example, the concept is applied to V-Fe alloy membrane [28]. As shown in Figure 14, the given temperature and pressure conditions are set to be 300 °C, 0.20 MPa at the feed side, and 0.01 MPa at the permeation side, respectively. These values are just an example case. Here, Fe is selected as an alloying element to control the PCT curve appropriately because the addition of Fe shifts the PCT curve largely to the upper left region [46]. Figure 14a shows the PCT curves of pure V [47] and V-2.5, V-7.5, V-10, and V-11 mol%Fe alloys at 300 °C. The addition of Fe shifts the PCT curve largely to the upper left region. Under the given condition, the hydrogen concentration in pure V, V-2.5 mol%Fe, and V-7.5 mol%Fe at the feed side exceeds the DBTC like the PCT curve (i) in Figure 13. For instance, the hydrogen pressure at the feed side needs to be reduced to 0.07 MPa when the V-7.5 mol%Fe is applied. The PCT curve for V-11 mol%Fe alloy is over shifted to the upper left region like the PCT curve (iii) in Figure 13. V-10 mol%Fe has the most appropriate hydrogen solubility, like the PCT curve (ii) shown in Figure 13. Figure 14b shows the PCT factors for V-7.5, V-10, and V-11 mol%Fe alloys estimated under the conditions represented by the star symbols in Figure 14a. Under the given condition, V-10 mol%Fe has the highest PCT factor under this condition. Figure 14c presents the time dependence of the hydrogen flux normalized by the inverse of the membrane thickness (*J·L*) for Pd-27 mol%Ag-coated V-10 mol%Fe alloy membrane measured at 300 °C. For comparison, the experimental results for V-7.5 Fe mol%Fe and V-11 mol%Fe alloy under the conditions represented by the star symbols in Figure 14a and the estimated value for Pd-23 mol% Ag alloy membranes under the same condition are given in the figure. The normalized hydrogen flux through V-7.5, V-10, and V-11 mol%Fe alloy membranes are approximately 4.0 × 10^−5^, 5.5 × 10^−5^, 4.7 × 10^−5^ mol H_2_ m^−1^·s^−1^, respectively. Among them, the normalized hydrogen flux through V-10 mol% Fe alloy membrane is the highest. It is noted here that the V-11 mol%Fe alloy membrane exhibits higher normalized hydrogen flux than V-7.5 mol% Fe alloy membrane although the PCT factor of the V-11 mol% Fe alloy is lower than that of V-7.5 mol% Fe alloy. This is because the addition of higher amount of Fe into V enhances the hydrogen mobility (*B*) at 300 °C in Equation (18) [28]. Additionally, V-10 mol% Fe alloy membrane exhibits approximately five times higher normalized hydrogen flux than the Pd-23 mol% Ag alloy membrane. The hydrogen flux is stable and almost constant for at least 1000 h. When the thickness of the V-10 mol% Fe alloy membrane is 50 μm, the hydrogen flux (*J*) is estimated to be about 75 mL·cm^−2^·min^−1^. After the hydrogen permeation test, hydrogen gas is evacuated, and the gas leak test is performed using helium gas. It is confirmed that there is no crack on the membrane due to hydrogen embrittlement. Furthermore, the membrane is deformed into a hat shape, indicating that the plastic deformation takes please even in the hydrogen atmosphere. These results demonstrate that the concept for alloy design shown in Figure 13 is effective for designing non-Pd-based alloy membranes with high hydrogen permeability and strong resistance to hydrogen embrittlement under given temperature and pressure conditions.

**Figure 15:**
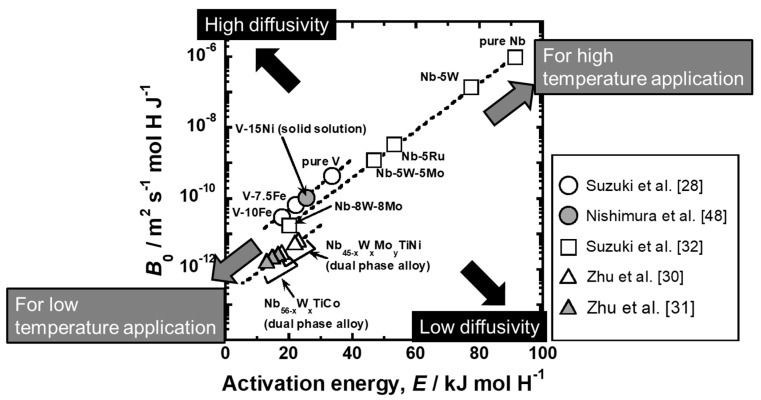
Relationship between the pre-exponential factor (*B*_0_) and the activation energy (*E*) for V-based solid solution alloys [28,48], Nb-based solid solution alloys [32], and Nb-based dual phase alloys [30,31].

The PCT factor (*f*_PCT_) can be enhanced based on the concept shown in Figure 13. On the other hand, the hydrogen flux is affected not only by *f*_PCT_ but also by the mobility of hydrogen atoms (*B*). The mobility of hydrogen atoms can be quantified from the slope of lines for the relationships between the hydrogen flux and *f*_PCT_, as described above (Figure 10d). As shown in Figure 11, the temperature dependence of the mobility of hydrogen atoms is described by the following Arrhenius equation.

(23) $$B=B_{0}\exp\left(-\frac{E}{RT}\right),$$

where *E* is the activation energy for hydrogen diffusion and *B*_0_ is the pre-exponential factor. Lower activation energy and higher pre-exponential factor lead to higher hydrogen diffusivity. The pre-exponential factor and the activation energy for V-based solid solution alloys [28,48], Nb-based solid solution alloys [32], Nb-based dual phase alloys [30,31] are analyzed and shown in Figure 15 (*B*_0_-*E* map). If the plots are located closer to the upper left region in the figure, higher hydrogen diffusivity is expected. It is interesting that there is positive linear correlation between the pre-exponential factor and the activation energy for each alloy system. Such a linear correlation observed in Arrhenius plot is known as Meyer-Neldel rule [49]. Due to Meyer-Neldel rule, the plots are located from the right upper region to left lower region. The materials located on the upper right region, e.g., pure Nb and Nb-5W, exhibits higher hydrogen diffusivity than the materials located on the lower left region at high temperature and are suitable for being utilized at high temperature applications. On the other hand, the materials located on the lower left region, e.g., V-10 mol%Fe, Nb-8 mol%W-8 mol%Mo and Nb_56-x_W_x_TiNi alloys, show higher hydrogen diffusivity than the materials located on the upper right region at low temperature and are preferred to low temperature applications. In addition, when systems (V- or Nb-based) or microstructures (single solid solution or dual phase) are different, the data points seem to be located on different lines. The data points of Nb-based dual phase alloys with different secondary phases align on almost the same line. Among the materials shown in Figure 15, V-based solid solution alloys seem to have an advantage for hydrogen-permeable alloy membrane in view of hydrogen diffusivity.

## 4. Summary

The methodologies for analyzing hydrogen permeability are reviewed, and the consistent descriptions of hydrogen permeation through dense metallic membranes were described in detail. These consistent descriptions of hydrogen permeability provide a way to analyze the hydrogen permeability consistently even under conditions of non-dilute hydrogen concentration. With the methods, the hydrogen flux can be estimated precisely under given conditions. In addition, the hydrogen permeation coefficient for the power law with different *n* can be compared consistently. The analysis based on the consistent description reveals that the anomalous temperature dependence of hydrogen permeability for Pd-Ag alloys is caused by the deviation of hydrogen solubility from Sieverts’ law. The PCT factor, which is linked directly with the shape of the PCT curve, provides an important suggestion for the design of not only Pd-based alloy but also non-Pd-based alloy membranes: the steep slope of PCT curves enhance the hydrogen flux through the membrane. The designed V-Fe alloy membrane, which has an appropriate PCT curve under a give condition, exhibits high and stable hydrogen flux for an extended period. In addition, a map for the mobility of hydrogen atoms is provided. The materials should be selected according to the operation temperatures and where the pre-exponential factor (*B*_0_) and the activation energy (*E*) are located on the map. The materials at the upper right region in the map (high *B*_0_ and *E*) should be used for high temperature applications, while the materials at left lower region in the map (low *B*_0_ and *E*) should be applied for low temperature applications.
